# Genome Sequence of *Mycobacterium tuberculosis* C2, a Cerebrospinal Fluid Clinical Isolate from Central India

**DOI:** 10.1128/genomeA.00842-14

**Published:** 2014-08-21

**Authors:** Rajpal S. Kashyap, Shradha S. Bhullar, Ravi P. More, Sampada Puranik, Hemant J. Purohit, Girdhar M. Taori, Hatim F. Daginawala

**Affiliations:** a Biochemistry Research Laboratory, Central India Institute of Medical Sciences (CIIMS), Bajaj Nagar, Nagpur, India; b Environmental Genomic Division, CSIR-National Environmental Engineering Research Institute (NEERI), Nehru Marg, Nagpur, India

## Abstract

We report the annotated genome sequence of a *Mycobacterium tuberculosis* clinical isolate from the cerebrospinal fluid of a tuberculous meningitis patient admitted to the Central India Institute of Medical Sciences, Nagpur, India.

## GENOME ANNOUNCEMENT

Tuberculosis (TB) is a major infectious disease that kills millions of people, mostly in developing countries like India (1). Among tubercular infections, tuberculous meningitis is the most common form of neurotuberculosis caused by *Mycobacterium tuberculosis* (*M. tuberculosis*) bacilli and the fifth most common form of extrapulmonary TB (2–4). The identification of sequence diversity in *M. tuberculosis* would provide a basis for understanding pathogenesis, immune mechanisms, and bacterial evolution. In earlier studies, isolation of different variants from different infected sites of patients has helped in the understanding of variability in *M. tuberculosis* in a wide clinical scenario (5). Furthermore, significant differences in the genome of the organisms have been documented among different clinical isolates (6).

Here we report the annotated genome sequence of a *M. tuberculosis* C2 clinical isolate from the cerebrospinal fluid (CSF) received by the pathology laboratory of the Central India Institute of Medical Sciences, Nagpur, India. The routine analysis of CSF was performed in the pathology laboratory and was then transferred to BacT/Alert 3D bottles for cultivation of the organism. In brief, CSF was pelleted and suspended in MP bottles containing Middlebrook 7H11 medium. The bottles were loaded into a BacT/Alert 3D machine (bioMérieux, Inc, Durham, NC) and incubated at 37°C for 42 days as per the manufacturer’s instructions. After checking for the purity of the culture by microscopy, the total DNA was isolated and the remaining culture was destroyed as per biohazard norms. The Institutional ethics committee of Central India Institute of Medical Sciences, Nagpur approved the study. Informed consent of the participating subject was obtained.

The whole-genome shotgun sequencing of *M. tuberculosis* C2 was performed on an Illumina MiSeq platform, which generated 774 MB raw reads sequence data, resulting in more than 100× sequencing coverage. A total of 1,690,781 high-quality reads were *de novo* assembled into 224 contigs, using GS Assembler/CLC genomics workbench version 6.0. In order to obtain a functional annotation of the genome, all the assembled contigs were annotated by the NCBI Prokaryotic Genome Annotation Pipeline (released 2013). The draft genome size of this strain is 4,377,597 bp with a G+C content of 65.5%. The genome possesses a total of 4,031 genes, including 3,935 coding sequences (CDSs), 53 RNAs, and 43 pseudo genes. Also SEED-based subsystem classification through Rapid Annotation using Subsystem Technology (RAST) (RAST Genome ID: 6666666.70925) analysis revealed that the isolate C2 showed *M. tuberculosis* NCGM2209 as the closest phylogenetic neighbor (score: 429) (7). In addition, to obtain functional genome relatedness, a total of 3,981 unigenes were predicted from contigs using a Prodigal microbial gene finding program (8). Further, the functional annotation was performed by aligning the unigenes to the non-redundant database of NCBI using BLASTx with e-value less than 1e−6 against the nr database (9). The analysis suggested that 93.33% CDS showed a high level of sequence similarity to *M. tuberculosis*; of which 40% CDS corresponds to *M. tuberculosis* H37Rv. The *M. tuberculosis* C2 genome carries multiple genes potentially involved in toxins and showed homology with *M. tuberculosis* H37Rv. A few hits from *M. bovis* and *M. marinum* have also been observed.

### Nucleotide sequence accession numbers.

This whole-genome shotgun project has been deposited at DDBJ/EMBL/GenBank under the accession no. JMEK00000000, and consists of contig sequences JMEK01000001 to JMEK01000224. The version described in this paper is version JMEK00000000.1.
